# Prevalence of Intestinal Protozoan Parasites and Associated Risk Factors among School Children in Merhabete District, Central Ethiopia

**DOI:** 10.1155/2021/9916456

**Published:** 2021-11-25

**Authors:** Nigus Dagne, Amir Alelign

**Affiliations:** ^1^Debre Birhan University, College of Natural and Computational Sciences, Department of Biology, Debre Birhan, Ethiopia; ^2^University of Gondar, College of Natural and Computational Sciences, Department of Biology, Gondar, Ethiopia

## Abstract

**Background:**

Intestinal protozoan parasitic infections remain one of the major public health problems in tropical regions especially, among developing countries such as Ethiopia. However, no enough epidemiological data is available in this respect in many parts of the country. Hence, this study was aimed at assessing the prevalence of intestinal protozoan parasitic infections and their associated risk factors among school children in Zeita village, Merhabete District, North Shewa Zone, Central Ethiopia.

**Methods:**

A cross-sectional parasitological survey was conducted from January to March 2020. A total of 280 children from Zeita primary school were selected using stratified random sampling techniques. Stool samples were collected and examined using direct wet mount, Formal-Ether concentration and Modified Zeihel-Neelsen staining techniques.

**Results:**

The overall prevalence of intestinal protozoan parasitic infection was found to be 46.8% (131/280). The three predominant protozoan parasites detected in this study were *Entamoeba histolytica*, *Giardia lamblia*, and *Cryptosporidium parvum* which were observed in 70 (25.2%), 54 (19.3%), and 7 (2.5%) of the study participants, respectively. Sociodemographic characteristics of the study subjects such as family occupation (*P* = 0.028), sources of drinking water (*P* = 0.001), water handling practice (*P* = 0.027), habit of eating vegetable (*P* = 0.001), and presence of latrine were observed to be significantly associated risk factors for the occurrence of human intestinal protozoan parasites.

**Conclusion:**

A high prevalence of intestinal protozoan parasitic infection which has been contributed by different risk factors was revealed in this study. The findings suggested a need of collaborative effort among the educational and health authorities to control the infection in the study area.

## 1. Introduction

Human intestinal parasites are identified as causes of morbidity and mortality throughout the world, particularly in underdeveloped countries [1]. A high prevalence of intestinal parasitic infections in human is positively correlated with poverty and poor environmental hygiene, lack of safe water supply, contamination of the environment by human excreta and animal wastes, and poor personal hygiene and living conditions [2].

Intestinal protozoan parasitic infections (IPPIs) are one of the major public health problems in tropical regions, especially among poor communities [3]. The most common intestinal protozoan parasites are *Entamoeba histolytica*, *Giardia lamblia*, and *Cryptosporidium species*. The diseases caused by these intestinal protozoan parasites are known as *amoebiasis*, *giardiasis*, and *cryptosporidiosis*, respectively, and they are associated with diarrhoea [4].

Intestinal protozoa are transmitted by the fecal-oral route and tend to exhibit similar life cycles consisting of oocyst and trophozoite stages. Fecal-oral transmission involves the ingestion of food or water contaminated with oocysts. In general, situations involving close human-human contact and unhygienic conditions promote transmission [5]. School children are the most commonly affected group than the general population due to their typical hand-mouth activity, uncontrolled fecal activity, water contamination, and their immature immune system [6].

*Entamoeba histolytica* and *Giardia lamblia* are estimated to infect about 60 million and 200 million people worldwide, respectively [7]. Reports from different parts of Ethiopia showed that there were differences in the prevalence rates of *amoebiasis*, *giardiasis*, and *cryptosporidiosis* [8–10].

In most parts of Ethiopia, no adequate epidemiological data is available, particularly in most vulnerable groups of the society such as school children. Therefore, the present study was conducted to determine the prevalence of intestinal protozoa parasitic infections and their associated risk factors among school children in Zeita village, Merhabete district, North Shewa Zone, Amhara Region, Central Ethiopia.

## 2. Materials and Methods

### 2.1. Description of the Study Area

A cross-sectional study was conducted from January to March 2020 in Zeita primary school, the only school available in Zeita village, located in Merhabete district, North Shewa zone, Amhara region, Central Ethiopia. Zeita is located at 202 kms north of Addis Ababa, the capital city of Ethiopia, with a total population of 5316 [11]. The elevation was approximately 1600-1800 meters above sea level with a mean annual temperature of 20°C. The area receives an average annual rainfall of approximately 1300-1600 mm. The inhabitants livelihoods based on a subsistent mixed farming system. Only governmental health facilities are available in the village. The village depends mainly on rivers and streams as sources of all-purpose water.

### 2.2. Sample Size and Sampling Techniques

The sample size was determined using a single population proportion formula described elsewhere [12–14]; *n* = *Z*^2^ × *p* (1 − *p*)/*d*^2^.

Where *Z* = 1.96 at 95% confidence interval, a prevalence (*p*) of 50% was considered due to lack of data from similar studies in the study area, and a 5% margin of error (*d* = 0.05) = 1.96^2^ × 0.50 × 0.50/0.05^2^ = 384. By considering a 10% (38) nonresponse rate, the total sample size was calculated to be 422. However, due to school absenteeism of students at the time of coronavirus disease 2019 (COVID-19) pandemic, we managed to collect data only from 280 respondents.

To achieve the total sample size, the children were stratified according to their age groups (5-10 years and >10 years) and then the sample study participants were selected randomly with equal proportion of grades and classes.

The present study included children of 1-8 grade level who were enrolled at Zeita Primary school in the 2019/2020 academic year. The inclusion criteria were being permanent resident in the study area at least for the previous 1 month before data collection; volunteers (both children and parents or guardians) to participate in the study. Children who took antihelminthic or antiprotozoa drugs within the last 3 months were excluded from participating in the study.

### 2.3. Data Collection

#### 2.3.1. Sociodemographic Data

Information on the subject's sex, age, and other sociodemographic factors including source of drinking water at home (protected or unprotected), water handling practices, presence or absence of latrines at their homes and school, latrine type, habit of washing of vegetables or fruits before eating, habit of eating raw vegetables, contact with animals, level of knowledge on personal hygiene practice, and environmental sanitation were collected using structured questionnaire. The questionnaire was prepared in English, and it was translated into the local language, Amharic. Then, the response was translated back to English.

#### 2.3.2. Stool Examination

The study participants were provided a labeled and leak-proof container, toilet paper, and applicator stick and were informed to put about 3 gm of stool using the applicator sticks. Fresh stool sample was collected from each consented study participant. Direct saline and iodine wet mount preparations were examined within 30 min after collection. Temporary microscopy station was set in the school compound for this purpose. The smears stained with modified Ziehl-Neelsen (MZN) method were considered with microscope to identify *Cryptosporidium* oocysts. The remaining sample was preserved with 10% formalin and transported to Alem ketema Enat Hospital laboratory. Then, all transported samples were processed and examined using the formol-ether concentration technique following the standard protocol explained elsewhere [15].

### 2.4. Data Analysis

All collected data were analyzed using SPSS Software version 20. Descriptive statistics were used to describe patient characteristics. Chi-square (*χ*^2^) test and logistic regression analyses were used to determine possible associations between infection and exposure to different risk factors. Values were considered statistically significant when the *P* value was found to be less than or equal to 0.05.

Considerations of some terms in the context of this study.

Abdominal pain: recurrent abdominal pain that can be caused by protozoan infection among children.

Personal hygiene of children: hand washing behavior of children both at home and school, particularly, after defecation.

## 3. Results

### 3.1. Sociodemographic Characteristics of the Study Participants

In the present study, a total of 280 school children (140 males and 140 females) were selected using stratified random sampling. The age range of the study population lies between 5 and 15 years. Out of the total 280 respondents, 127 (45.3%) and 153 (54.7%) had a family size of ≤4 and >4 persons, respectively. Regarding their parents' level of education, 129 (46.07%) of the study participants' parents were illiterates (unable to read and write). From a total of 280 study participants, about 24.2% of the respondents reported that they had always abdominal pain, whereas about 39.5% of them had never felt such symptoms. In addition, about 50.5% of the respondents were with poor knowledge about personal hygiene and environmental sanitation (Table 1).

### 3.2. Prevalence of Intestinal Protozoan Parasites

The overall prevalence of intestinal protozoan parasitic infections (IPPIs) among all age groups of the study participants was 131 (46.8%). The prevalence of intestinal protozoan parasitic infection in the age group 5-10 years old was 53.8% and 55.5% in males and females, respectively (Table 2). A chi-square test of independence showed that gender and age groups of the study participants were not significantly associated with parasitic protozoan positivity (*P* = 0.54) (Table 2).

### 3.3. Common Intestinal Protozoan Parasitic Species Identified

In the present study, three common intestinal protozoan parasite species were identified in the examined stools of school children. These were *Entamoeba histolytica*, *Giardia lamblia*, and *Cryptosporidium parvum* with an overall prevalence of 25%, 19.3%, and 2.5%, respectively (Table 3). However, no *Cryptosporidium parvum* was detected in females in the age group of 5-10 years. Moreover, the study revealed nonsignificant differences between the types of protozoan parasites detected among males and females of different age groups (Table 3).

### 3.4. Factors Associated with Intestinal Protozoan Parasite Infection

Occupation was one of the risk factors for intestinal protozoan parasitic infection in the present study (Table 4). In our study, out of 248 school children who had an agro-farming practicing families, about 49.1% were found to be positive for intestinal protozoan parasites. However, only 29.5% of children from a nonagro-farming practicing family were infected by protozoan parasites. The logistic regression analysis revealed that the odds are more than two times higher that children from an agro-farming practicing families will get protozoan parasitic infections compared to those from a non-farming ones, in which the association was statistically significant, OR = 2.47; *P* = 0.028 (Table 4).

## 4. Discussion

The observed overall prevalence of intestinal protozoan parasites in the present study, which was 46.8%, was lower compared to the reports of other similar studies in other parts of Ethiopia such as 72.9% in Gondar (Azezo), 83% in Jimma, and 83.8% in South East of Lake Langaano [16–18]. On the other hand, the prevalence observed in this study was higher than a study conducted in Babile (27.2%) and Southern Ethiopia (7.1%) [19, 20]. The differences in the findings of the various studies can be explained by variations in geography, personal hygienic conditions of the study subjects, and the prevailing climatic and environmental conditions under consideration.

In Ethiopia, previous studies documented that *Entamoeba histolytica*, *Giardia lamblia*, and *Cryptosporidium species* were the most common protozoan intestinal parasites [9, 19, 21, 22]. Consistently, the current study revealed that *Entamoeba histolytica*, *Giardia lamblia*, and *Cryptosporidium parvum* were predominantly identified in the examined stools of the school children in their respective order. However, no significant differences were observed among the types of protozoan parasitic infections with age and sex of the school children.

The overall prevalence of *E. histolytica* in the present study (25%) was higher than the report of a previous study from Alem ketema town, Central Ethiopia, which was 16.7% [23]. However, it was lower than the one conducted in Tigray region (39.7%) [24]. The overall prevalence of *Gardia lamblia* infection (19.3%) in the present study was comparable to previous reports in different parts of the country [25, 26]. In contrast, a study conducted in eastern Ethiopia (Dire-Dawa) reported a higher prevalence (38%) of *G. lamblia* among school children. Unless prior infections were protective for giardiasis, one would expect to see a higher rate of giardiasis in developing countries where the living standard of the society is very low [26]. The present study also revealed a total prevalence of *Cryptosporidium parvum* to be 2.5%, which is lower than the 9.4% prevalence detected earlier in central Ethiopia [27]. The variation in prevalence depends on factors such as geographical area; urban or rural setting of the society, the age group composition, and the socioeconomic conditions of the study subjects [28].

In each age group of the study participants, there was no statistically significance difference in the prevalence of *Cryptosporidium species* infections between males and females (*P* > 0.05). However, a higher prevalence of *Cryptosporidium species* infections was observed in the age group ≥10 years in females, which was consistent with another previous study [29]. The females do milking of cattle and clean animal dung from resting places (along with mothers) and hence the observed higher prevalence rates could probably reflect direct and indirect exposure of female children to the sources of infection, particularly, contaminated feaces. The higher prevalence of *Cryptosporidium species* infection observed in female children could not only be the result of contaminated water and food, but their frequent contact with domestic animals during milking and cleaning animal quarters could also contribute to the high prevalence as compared to males who rarely practice the above activities in the community.

Drinking water source conditions showed significant variation in the prevalence of protozoan parasites among the school children. This finding was in agreement with what was reported by previous studies in the country and elsewhere [21, 30]. The possible explanation for this difference is due to differences in households drinking water management or due to water source-restricted infections. Some households probably practiced good drinking water treatment activities such as filtration or boiling before consumption.

The absence of latrines both at schools and residential areas was observed to have a significant contribution to acquiring protozoan parasitic infections among children. Under poor hygienic conditions, feces and urine often enter the water body due to lack of proper latrine, and this enhances the transmission probability of intestinal parasites like protozoans and soil-transmitted disease through indiscriminate defecation habits [31]. In the present study, a significant association was found between intestinal protozoan parasitic infections and contact with animals and their faces. This report was in agreement with a previous study in Ethiopia [32]. This was probably due to contamination of animals and their faces with parasite egg larvae and oocysts.

Significant association was found between intestinal protozoan parasitic infections and knowledge of participants on personal hygiene practices and environmental sanitation. Study participants who had poor knowledge to personal hygiene practice and environmental sanitation were more likely to acquire intestinal protozoan parasite infections (56.7%) compared with those who had good knowledge of personal hygiene practices and environmental sanitation (36.8%). This report was in agreement with a previous study [33]. This may be due to unaware children do not take care of their personal hygiene and environmental sanitation. Usually, children play in contaminated outdoor environments, in and around disposal sites and do not practice personal hygiene activities such as washing hands before and after meals [34], which can certainly cause serious health problems including IPPI.

## 5. Conclusion

The findings of the current study showed that intestinal protozoan parasitic infection was highly prevalent and remains an important health problem among Zeita Primary school children with *Entamoeba histolytica*, *Giardia lamblia*, and *Cryptospordium species* were the most common human intestinal protozoan parasite species identified. An increasing prevalence of intestinal protozoan parasite infection in this study was associated with factors such as sources of drinking water, toilet availability, water handling practices, eating habits, knowledge about personal hygiene practice, and environmental sanitation among school children. Hence, a collaborative effort between district health departments and the school health programs is needed to promote the availability of safe and protected water supply and toilet facilities both at schools and residential areas. Moreover, delivering health education for school children about personal hygiene practices and environmental sanitation transmission and prevention of human intestinal protozoan parasite infections is vital in the control of such public health threats.

### 5.1. Limitations

Due to the current COVID-19 pandemic and the subsequent school absenteeism of children, the present study was limited to collect a sufficient amount of data. Moreover, the study failed to diagnose multiple infections of intestinal protozoan parasites in the stools of participants.

## Figures and Tables

**Table 1:** Sociodemographic characteristics of study participants in Zeita Primary School, Merhabete district, Amhara region, Central Ethiopia, 2020 (*N* = 280).

Character	Frequency	Percentage
Sex		
Male	140	50.0
Female	140	50.0
Age groups (years)		
5-10	116	41.5
>10	164	58.5
Family size (individuals)		
≤4	127	45.4
>4	153	54.6
Parents' literacy		
No basic education	129	46.1
Basic education	63	22.5
High school education	57	20.4
Tertiary education	31	11.1
Abdominal pain		
Always	68	24.2
Sometimes	101	36.1
Never	111	39.7
Knowledge of children to personal hygiene practice and environmental sanitation		
Poor	141	50.4
Good	139	49.6
Total	280	100

*N*: total number of study participants.

**Table 2:** Prevalence of intestinal protozoan parasitic infections by age and sex among school children in Merhabete district, Amhara region, Central Ethiopia, 2020 (*N* = 280).

Age group (in years)	Male		Female		Both sexes		*χ* ^2^	*P* value
	No. examined	No. positive (%)	No. examined	No. positive (%)	No. examined	No. positive (%)		
5-10	57	30 (53.8)	59	33 (55.3)	116	63 (22.5)	0.37	0.542
>10	83	36 (43.4)	81	32 (39.5)	164	68 (41.5)		
Total	140	66 (47.4)	140	65 (46.4)	280	131 (46.8)	0.042	0.838

*χ* ^2^: Chi-square.

**Table 3:** Common intestinal protozoan parasite species identified from examined school children in Merhabete district, Amhara region, Central Ethiopia, 2020.

Age group and sex	Total no. examined	Protozoan parasites species		
		*Eh*	*Gl*	*Cp*
5-10 years				
Male	57	15 (26.6)	13 (23)	2 (3.8)
Female	59	18 (30.8)	15 (24.6)	0 (0)
*χ*^2^		0.130	0.066	NA
*P* value		0.708	0.796	NA
>10 years				
Male	83	21 (25.3)	13 (15.7)	1 (1.2)
Female	81	16 (19.8)	13 (16.0)	4 (4.9)
*χ*^2^		0.456	0.003	1.818
*P* value		0.499	0.954	0.177
Total	280	70 (25)	54 (19.3)	7 (2.5)

*Eh*: *Entamoeba histolytica*; *Gl*: *Giardia lamblia*; *Cp*: *Cryptosporidium parvum*; NA: not applicable.

**Table 4:** Association of sociodemographic risk factors with intestinal protozoan parasite infection of school children in Merhabete district, Central Ethiopia, 2020.

Characters	Total	Intestinal protozoan parasite infection	OR (95% CI)	*P* value
		Positive		
Family occupation:				
Agro-farming	248	122 (49.1)	2.47 (1.100-5.561)	0.028
Nonfarming	32	9 (29.5)		

Parent education level				
Illiterate	102	54 (52.9)		
Literate	178	78 (43.8)	1.44 (0.884-2.351)	0.141

Presence of latrine at home				0.037
Yes	99	38 (38.4)	0.59 (0.357-0.970)	
No	181	93 (51.4)		

Latrine type (*n* = 99):				0.713
Pit latrine with cover	42	17 (40.4)	1.17 (0.514-2.641)	
Pit latrine without cover	57	21 (36.8)		

Source of drinking water				0.001
Protected (pump water)	94	30 (31.7)	0.39 (0.234-0.664)	
Unprotected (river, pond borehole)	186	101 (54.5)		

Water handling				
Direct	204	104 (51)		
Boiling	12	4 (33.3)	0.018 (0.005-0.032)	0.027
Filtering	58	22 (37.9)		
Chemical treating	6	1 (16.67)		

Washing of vegetables or fruit before eating				
Always	144	57 (39.6)	0.265 (0.172-0.276)	0.136
Sometimes	80	42 (53.7)		
Do not wash	56	31 (55.9)		

Habits of eating raw vegetable				
Yes	182	98 (53.8)	2.29 (1.379-3.828)	0.001
No	98	33 (33.6)		

Contact with animals				
Yes	183	96 (52.5)	1.87 (1.129-3.094)	0.015
No	97	36 (36.8)		

Knowledge				
Poor	141	80 (56.7)	2.26 (1.400-3.655)	0.001
Good	139	51 (36.7)		

OR: odds ratio; CI: confidence interval.

## Data Availability

The datasets generated and/or analyzed during the current study are available from the corresponding author on reasonable request.

## Abbreviations

CI: Confidence interval
COVID-19: Coronavirus disease 2019
IPPIs: Intestinal protozoan parasitic infections
OR: Odds ratio
SPSS: Software package for social sciences.
